# Androgen Receptor Function Links Human Sexual Dimorphism to DNA Methylation

**DOI:** 10.1371/journal.pone.0073288

**Published:** 2013-09-04

**Authors:** Ole Ammerpohl, Susanne Bens, Mahesh Appari, Ralf Werner, Bernhard Korn, Stenvert L. S. Drop, Frans Verheijen, Yvonne van der Zwan, Trevor Bunch, Ieuan Hughes, Martine Cools, Felix G. Riepe, Olaf Hiort, Reiner Siebert, Paul-Martin Holterhus

**Affiliations:** 1 Institute of Human Genetics, Christian-Albrechts-University Kiel & University Hospital Schleswig-Holstein, Campus Kiel, Kiel, Germany; 2 Department of Pediatrics, Christian-Albrechts-University Kiel & University Hospital Schleswig-Holstein, Campus Kiel, Kiel, Germany; 3 Department of Pediatrics, University of Lübeck & University Hospital Schleswig-Holstein, Campus Lübeck, Lübeck, Germany; 4 Core Facility, Institute of Molecular Biology gGmbH, Mainz, Germany; 5 Department of Pediatrics, Division of Pediatric Endocrinology, ErasmusMC-Sophia, Rotterdam, The Netherlands; 6 Department of Clinical Genetics, ErasmusMC, Rotterdam, The Netherlands; 7 Department of Paediatrics, Addenbrooke’s Hospital, University of Cambridge, Cambridge, United Kingdom; 8 Department of Pediatrics, University Hospital Gent, Gent, Belgium; RWTH Aachen University Medical School, Germany

## Abstract

Sex differences are well known to be determinants of development, health and disease. Epigenetic mechanisms are also known to differ between men and women through X-inactivation in females. We hypothesized that epigenetic sex differences may also result from sex hormone functions, in particular from long-lasting androgen programming. We aimed at investigating whether inactivation of the androgen receptor, the key regulator of normal male sex development, is associated with differences of the patterns of DNA methylation marks in genital tissues. To this end, we performed large scale array-based analysis of gene methylation profiles on genomic DNA from labioscrotal skin fibroblasts of 8 males and 26 individuals with androgen insensitivity syndrome (AIS) due to inactivating androgen receptor gene mutations. By this approach we identified differential methylation of 167 CpG loci representing 162 unique human genes. These were significantly enriched for androgen target genes and low CpG content promoter genes. Additional 75 genes showed a significant increase of heterogeneity of methylation in AIS compared to a high homogeneity in normal male controls. Our data show that normal and aberrant androgen receptor function is associated with distinct patterns of DNA-methylation marks in genital tissues. These findings support the concept that transcription factor binding to the DNA has an impact on the shape of the DNA methylome. These data which derived from a rare human model suggest that androgen programming of methylation marks contributes to sexual dimorphism in the human which might have considerable impact on the manifestation of sex-associated phenotypes and diseases.

## Introduction

A wealth of phenotypic dimorphisms differentiates males from females. While the most evident sexual dimorphism occurs in the genitalia, there are also many extra-genital sites of the body characterized by sexual dimorphisms of anatomy and function including sex specific differentiation of the brain [1]. Eventually, behavioral traits have a well-documented sex specificity already present in children [2], [3]. Various human diseases show sex-specific incidences, e.g., cancers like mantle cell lymphoma [4] and Burkitt lymphoma [5] as well as auto-immune diseases like Sjögren’s Syndrome [6] and systemic lupus erythematodes [7]. Biological sex also modifies risks for common disorders like asthma [8] and cardiovascular disease [9]. This indicates that sexual dimorphism is an important human phenotype modifier that can affect prevalence, course and severity of diseases [10].

Male or female biological sex is only initially determined by the presence or the absence of the *SRY*-gene on the Y-chromosome resulting in either testicular or ovarian development (i.e. gonadal sex). It is indeed the presence or absence of androgen (testosterone and dihydrotestosterone, respectively) activating the androgen receptor (AR) pathway between the 8^th^ to 13^th^ week of gestation which is the key determinant for establishing the male or the female phenotypic sex [11]. It is, however, still largely unknown, how androgens and the AR pathway implement human sexual dimorphisms.

At the cellular level, activation of the AR by androgen results in tissue-specific up – and down-regulation of gene transcription in androgen responsive tissues, e.g., in genital skin fibroblasts [12] or in the prostate [13]. In addition to this classical endocrine perspective of AR function a set of elegant experiments in rats has previously indicated a male androgen programming window in which the early prenatal androgen environment determines key features of the adult sexual phenotype like phallus size, anogenital distance and Sertoli-cell number. This program can only in part be modified by postnatal androgen supply [14], [15]. Interestingly, there is also increasing evidence from other animal studies showing that prenatal androgen programming may be an important modifier of extragenital health states and diseases, e.g., via CNS-programming of weight control [16], [17] or via androgen programming of liver function and insulin sensitivity [18]–[20]. Anway and coworkers have induced early prenatal disruption of the AR pathway in rat embryos by intraperitoneal injections of the anti-androgen vinclocolin in mother rats [21]–[24]. The authors have shown that this treatment has been associated with comprehensive changes of the embryonic F1 testis transcriptome including the DNA methyltransferase genes Dnmt3A and Dnmt3L. In addition, transcriptional changes also occurred in F2 - F3 generation animals indicating transgenerational epigenetic effects of this drug [24]. Moreover, adult F1 animals and subsequent generations F2 - F4 developed various disease states and tissue abnormalities including prostate disease, kidney disease, immune system abnormalities, reduced spermatogenic capacity and tumor development [22],[23]. These findings suggest long lasting and transgenerational impact of the embryonic AR pathway disruption. Intriguingly, comparable transgenerational changes of gene transcription in response to vinclocolin have been observed in the rat brain transcriptome being accompanied by behavioral changes in respective animals [25].

In contrast to animals it is much more difficult to prove androgen programming in humans since experimental hormonal interventions are not possible for ethical reasons. Moreover, since males and females always differ simultaneously in both, sex chromosomes and sex hormones, it is hardly possible to decipher their differential influences on sexual dimorphisms. Remarkably, unique natural human models to distinguish chromosomal and hormonal influences are disorders of sex development (DSD), in particular the androgen insensitivity syndrome (AIS) in which inactivating mutations in the AR-gene abolish the AR-signaling pathway. This leads to impaired masculinization in genetically male individuals carrying a 46,XY karyotype. AIS-phenotypes range from complete AIS (CAIS) with normal female external genitalia through partially virilized (ambiguous) genitalia (PAIS) to minimal AIS (MAIS) with only infertility in otherwise normal males depending on the residual AR function [26]. Using AIS as a model, we showed previously by microarray analyses at the transcriptome level that androgens were able to program long-term persisting gene transcription patterns in human tissues, e.g., in external genital fibroblasts [27], [28] as well as even outside the genitalia in peripheral blood mononuclear cells [29].

We here hypothesized that androgen programming in the human occurs at the epigenome level, especially by differential programming of DNA-methylation states. While there is increasing evidence for the role of epigenetics in tissue differentiation and disease modification [30], [31], the potential role of epigenetics in shaping sexual dimorphisms and particularly the involvement of sex hormones therein are hardly understood. Up to date, X-inactivation is the major example of sex-specific epigenetic differences between males and females in humans but it depends on the number of X-chromosomes rather than presence or absence of sex hormones, namely androgens [32], [33]. We here show by microarray based methylation profiling that 46,XY individuals with an AR pathway disruption due to inactivating AR mutations have significant changes of their epigenomic signature in genital fibroblasts compared with normal males and intact AR. Therefore, our experiments suggest that androgen programming in the human results in changes in the epigenome.

## Methods

### Cell Culture

The study was approved by the ethical committee of the Christian-Albrechts-University of Kiel (CAU), Germany (D410/08). For samples included in the study which were obtained in the framework of the Euro DSD project (FP7 project 201444 from 2008 to 2011) written informed consent has been obtained in accordance with the protocols approved by the ethical commitee of the Christian Albrechts-University of Kiel (AZ: D410/08; http://www.eurodsd.eu/en/ethics.php). For fibroblast cultures established before Euro DSD, verbal informed consent was obtained by the surgeons operating the patient and taking the biopsy according to ethical approval and in line with respective institutional guidelines. A form documenting information and consent in the patients’ clinical files was provided according to ethical approval. Cell culture of 46,XY primary fibroblasts from scrotum/labia majora from donors with PAIS (n = 13), MAIS (n = 1) or CAIS (n = 12) and 8 male controls as well as isolation of genomic DNA were performed as detailed in [4]. The definition “control” in this study is based on the clinical documentation of a completely normal male external genital phenotype (i.e., complete androgen-mediated fusion of the male genital midline) as opposed to the incomplete or absent external genital virilization in the PAIS and the CAIS patients, respectively. The one MAIS patient had originally been assigned to the normal controls due to a normal male phenotype of the external genitalia (“S8”). However, our previous functional characterization of this fibroblast strain had revealed impaired androgen-mediated induction of the AR target gene *APOD* [12]. Subsequent sequence analysis of the *AR* gene showed that this person indeed carried a p.I841S *AR* gene mutation [12] and thus suffered from a mild form of AIS (MAIS) with the clinical symptom of isolated male infertility. Therefore, this sample was allocated as AIS sample despite normal male external virilization for analyses in this study. Mutational analyses in the remaining normal male controls (S3, S4, S5, S9, S11, S12, S13, S15) did not reveal AR gene mutations. Complete data are available at Gene Expression Omnibus (GSE31362).

### DNA Methylation Analysis

DNA methylation analysis using HumanMethylation27 BeadChips was performed as described [34], [35]. 13 AIS samples and all controls were analyzed in duplicates. Samples with gene call rates <95% as analyzed with BeadStudio software (Illumina, Inc.) and CpGs with detection p-values >0.05 in at least one experiment were excluded from analyses. 54 hybridizations with 25,988 CpGs entered the analysis.

Hierarchical cluster analyses based on average beta-values were performed using OMICS Explorer (v.2.1(25); Qlucore, Sweden). Reproducibility and accuracy of results obtained with the HumanMethylation27 BeadChip have been shown by us and others before [35]. Single nucleotide polymorphisms putatively interfering with DNA methylation analysis were identified using information provided by Illumina (https://my.illumina.com/myillumina/bulletin/K0igwwHjyE-5J35-sPKU2A/using-humanmethylation450-and-understanding-underl). Polymorphisms with an allele frequency >5% in the population which are located within a distance of 3 bp next to the cytosine analyzed on the array were identified (Tables S2 and S3 (“SNP”)). The Venny software package was used to generate venn diagrams [36].

### Analysis of Enrichment for Promoter Classes in Differentially Methylated Genes, Imprinted Genes and AR Target Genes

Proportions of imprinted genes, AR target genes and promoter classes in the particular groups of genes and the genes present on the HumanMethylation27 Bead Chip were compared using the χ^2^ test (two-sided; Prism, ver. 4.02). Relative risks and odds ratios were calculated using Prism.

Information on imprinted genes was taken from Luedi et al. [37] as well as on freely accessible databases (http://igc.otago.ac.nz and http://www.geneimprint.com).

We used a previously suggested classification into promoters with high (HCP), and low (LCP) CpG content promoter regions to determine whether differentially methylated genes are characterized by different CpG compositions [38]. Information whether CpG loci present on the array were located in a CpG island was available from Illumina. For enrichment analysis of putative AR-target genes in the group of differentially methylated genes as well as of the genes present on the array GATHER has been utilized (http://gather.genome.duke.edu [39]).

### Analysis of Variability in the DNA Methylation Pattern

Here we focused on CpG with high variability in the AIS samples but low variability in the controls. Therefore, CpGs characterized by differences in their beta-values ranging in control samples up to 0.34 and differences of the interquartile differences above 0.16 between controls and AIS samples were considered as being variably methylated in AIS. A deduction of these parameters is detailed in the supplement (Methods S1). Applying an analogous approach we determined loci showing high variability in normal controls but not in AIS samples.

## Results

### Identification of DNA Methylation Patterns in Genital Skin Fibroblasts of AIS Individuals

To provide insights on how androgen signaling may epigenetically imprint human external genitalia derived cells independent of the sex chromosomal composition, we have quantified the DNA-methylation levels of 27,568 CpGs in cultured human skin fibroblasts from the labioscrotal folds of individuals with molecular proven AIS and different degrees of impaired external genital virilization (n = 26) using an array based assay. Fibroblasts were obtained from 12 CAIS individuals, 13 PAIS individuals, one MAIS individual and 8 males with phenotypic normal male external genitalia. Molecular analysis of the *AR* gene revealed protein changing mutations in 24 AIS individuals. In two individuals with AIS but no detectable protein changing mutations biochemical analyses indicated impaired AR function (for details see supplementary Table S1) [12], [27]. Methylation values were compared to those of males with phenotypic normal male external genitalia. 25,988 CpG loci passed the quality tests. Duplicate samples showed good concordance (r^2^-range 0.9874–0.9964).

**Figure 1:**
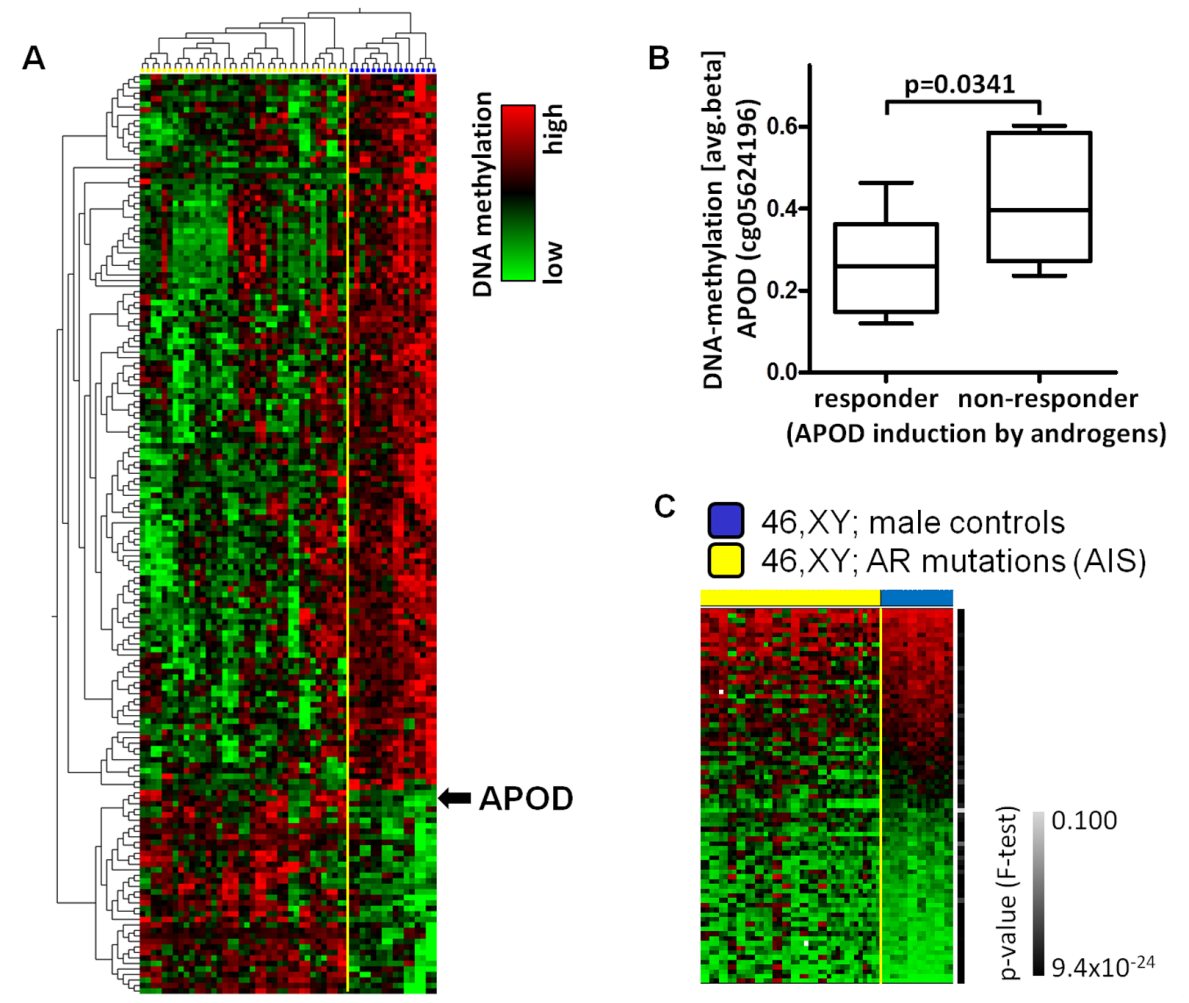
Array-based DNA methylation analysis of 26 AIS genital fibroblasts and 8 male control genital fibroblasts. (A) Supervised cluster analysis of DNA-methylation data obtained from genital fibroblasts separates individuals with AIS (yellow) from male controls (blue) (q<0.04). DNA-methylation is presented on a relative scale. To demonstrate reproducibility all hybridizations performed in duplicates are shown separately. (B) DNA-methylation of APOD (cg05624196) in fibroblasts lacking induction of APOD upon androgen treatment (non-responder) was significantly higher compared to responding fibroblasts (responder). (C) Variability in the DNA-methylation in AIS (yellow) compared to male controls (blue). Green: low, black: medium, red: high avg-beta values. The right bar indicates p-value (F-test).

To identify CpG loci differentially methylated between AIS samples and controls we performed a differential methylation analysis (Figure 1a, FDR<0.04, t-test). By this approach, a total of 167 CpG loci representing 162 unique human genes showed significantly differential methylation in AIS as compared to the controls. Of these, 36 genes (22%) were hypermethylated in AIS samples while the majority of 126 genes (78%) were hypomethylated in AIS samples. These data showed that the absence of intact AR-signaling in genital fibroblasts of XY individuals with AIS is associated with significant changes of DNA methylation.

### Genes Aberrantly Methylated in AIS are Characterized by Low CpG Content Promoters

**Figure 2:**
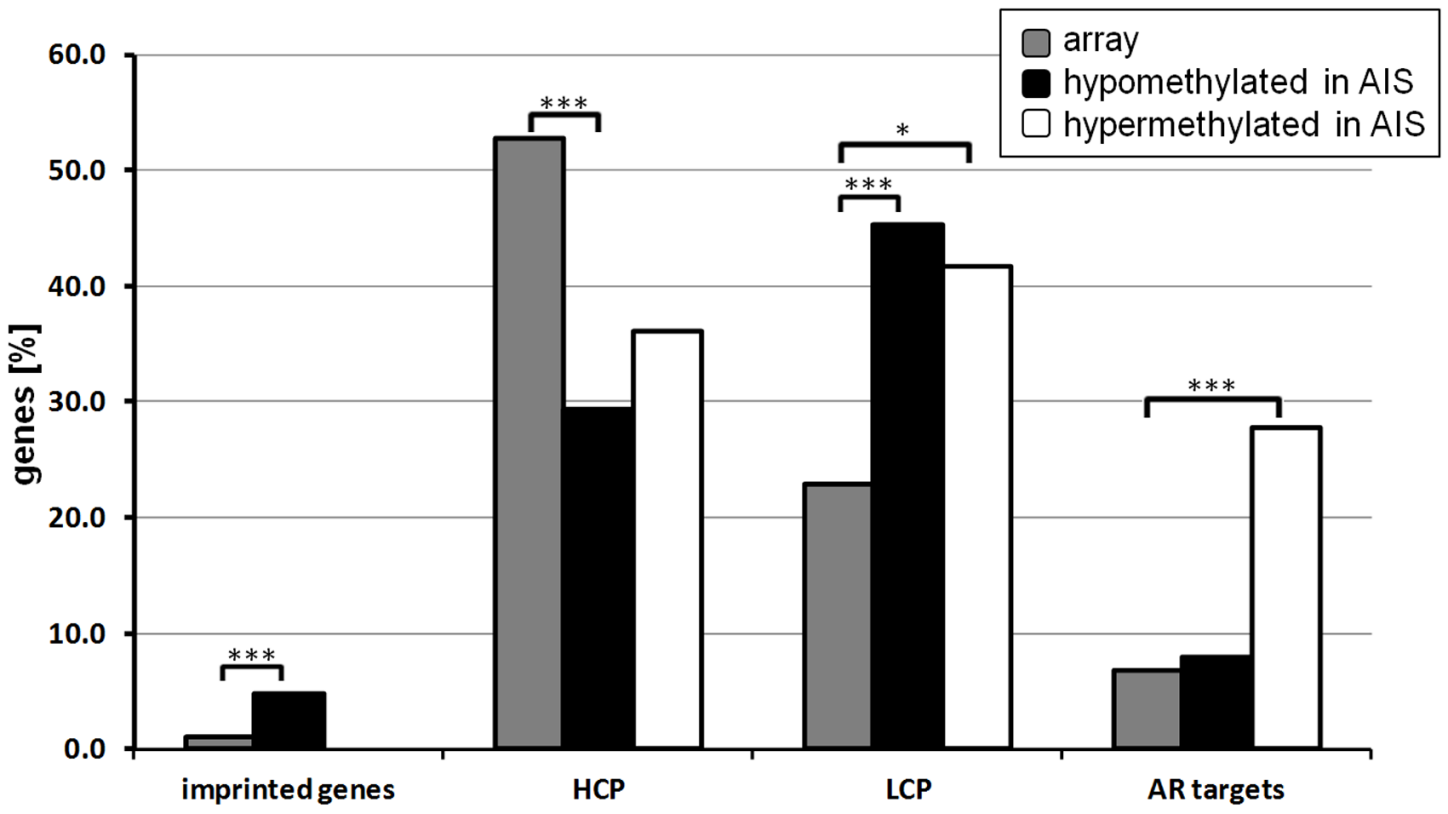
Enrichment of genes either hypo- or hypermethylated in AIS as compared to normal controls. The bar plots show percentage of loci either located in imprinted genes, genes containing either promoters with high CpG (HCP) or low CpG (LCP) content as well as the percentage of AR target genes as determined by the GATHER tool. Grey bars: percentage of genes present on the array, black bars: percentage of genes hypomethylated and white bars: percentage of genes hypermethylated in AIS patients. p-values have been determined applying χ^2^-test.

To further characterize the aberrantly methylated genes in AIS we have first classified the genes according to their promoter CpG content into high CpG (HCP) and low CpG (LCP) promoter genes (34). The genes found hypomethylated in AIS as compared to controls were significantly enriched for genes characterized by low CpG content promoters (LCP, p<0.0001, OR = 2.78, RR = 1.98) while they were depleted for genes with high CpG content promoters (HCP, p<0.0001, OR = 0.37, RR = 0.56, Figure 2). Interestingly, genes found hypermethylated in AIS as compared to controls were also enriched for genes with low CpG content promoters (LCP, p<0.05, OR = 2.41, RR = 1.82). These results suggest that genes with low CpG content promoters become preferentially aberrantly methylated in AIS as compared to controls.

### Genes Hypomethylated in AIS are Enriched for Genes Regulated by Genomic Imprinting

Moreover, to determine the impact of paternal imprinting, we identified known imprinted genes [37] in the group of aberrantly methylated genes in AIS. The genes found hypomethylated in AIS were significantly enriched for genes regulated by genomic imprinting (p = 0.0006, OR = 3.463, Figure 2). The imprinted genes included *GNAS, MEST, PEG3*.

### AR Target Genes are Aberrantly Methylated in AIS

We wondered whether due to the decreased AR activity, genes regulated by AR were particularly susceptible to aberrant DNA methylation in AIS as compared to the controls. Remarkably, the genes hypermethylated in AIS were significantly enriched for AR target genes identified by the GATHER tool [39] (p<0.0001, OR = 5.23, RR = 4.06) (Figure 2, Table S2, Figure S1) while hypomethylated genes were not enriched for AR targets (OR = 1.17, RR = 1.16). These findings support the idea that lack of AR binding to these genes due to AR mutations leads to the establishment of aberrant DNA methylation patterns in AIS.

### Low APOD Expression Inversely Correlates with DNA Methylation

Among the genes hypermethylated in AIS samples was *APOD* (Apolipoprotein D), which we have previously found to be strongly androgen-inducible in scrotal fibroblasts [12]. Interestingly, those AIS fibroblast strains that showed a significantly higher *APOD* methylation level at the transcription start (TSS) (chr3∶196,792,172; NCBI36/hg18, TSS10) as compared to male controls in our present study lacked androgen mediated *APOD* induction in a previous study [12] (Figure 1C). Since DNA methylation at the TSS correlates closely with gene repression [40] and furthermore this CpG is located within a known DNase I hypersensitive site [41], this might point to functional relevance of the methylation status for transcriptional control of *APOD* (Figure S2).

### Disruption of the AR Pathway in AIS Significantly Increases the Variability of DNA Methylation in a Set of Genes in Genital Skin Fibroblasts

By analyzing the DNA methylation data we noted a striking variability in the DNA methylation of numerous CpG loci in the AIS patients which was not observed in the normal male controls with intact AR. Consequently, we focused our analyses on systematic identification of the CpG loci with high variability in AIS samples and low variability in control samples and vice versa. In essence, we detected 79 CpG loci (corresponding to 75 genes; Table S3) with variable DNA-methylation in AIS, but homogenous DNA-methylation in controls. Vice versa, there was only a small set of genes (n = 3) with high variability of methylation in male controls, but homogeneous methylation in AIS (Figure 1C) (p<0.001). Thus, the number of genes with variable methylation was significantly higher in AIS than in controls. Remarkably, the list of genes differentially methylated between AIS and controls as identified by our first approach (see above) and the list of genes characterized by a high variability in DNA methylation in AIS overlapped in only 4 CpG loci (Figure S3). Interestingly, like the hypomethylated genes described above, the genes with variable DNA methylation in AIS were significantly enriched for genes with LCP (p<0.001, OR = 2.38, RR = 1.81) and depleted for genes with HCP (p<0.0001, OR = 0.37, RR = 0.56). There was no enrichment of known imprinted genes (n = 2) and, in particular, no enrichment of AR target genes (n = 0). To address the question whether genetic polymorphisms (SNPs) mimicked epigenetic variability causing misleading results, we identified CpG loci with a frequency >5% in the population which are located within a distance of 3 bp next to the cytosine analyzed on the array. Only 2 CpG loci (cg08008233 and cg12215675) fulfilled these criteria (Table S3). Thus, genetic variability can most likely be excluded as a major reason for the epigenetic variability found in AIS patients.

## Discussion

We here show that mutational disruption of one single nuclear hormone receptor pathway, the AR pathway in humans, leads to significant alterations of DNA methylation of numerous genes in cultured genital fibroblasts of 46,XY individuals with AIS compared with comparable cells from normal 46,XY males having an intact AR. Therefore, our data support the idea that androgens epigenetically program sexual dimorphism in the human by changing DNA methylation marks in the epigenome. Our data are well in accordance with our previous studies demonstrating long-term androgen programming of the transcriptomes in these cells [27], [28] and outside the genitalia in peripheral blood mononuclear cells [29].

Intriguingly, the methylation changes in AIS occur in two qualitatively distinct patterns comprising two different sets of genes with only 6 genes contributing to both groups. The first and most obvious pattern involves 162 genes differentially methylated between AIS and normal controls. The second pattern involves 75 genes and it is characterized by an overt and statistically significant increase of variability of DNA methylation in AIS which is not observed in male control cells with an intact AR.

One of the most striking observations when analyzing the gene set of the first pattern is that there is a significant enrichment of AR target genes among the genes hypermethylated in AIS. This observation might be explained by a current model suggesting that diminished gene activation (e.g. due to mutations in activating receptors) results subsequently in increased DNA methylation of the silenced genes [42]–[44]. This is supported by our functional analyses showing, that those AIS fibroblast strains that lacked androgen mediated APOD induction in a previous study [12] showed a significantly higher *APOD* promoter methylation level as compared to male controls in our present study.

**Figure 3:**
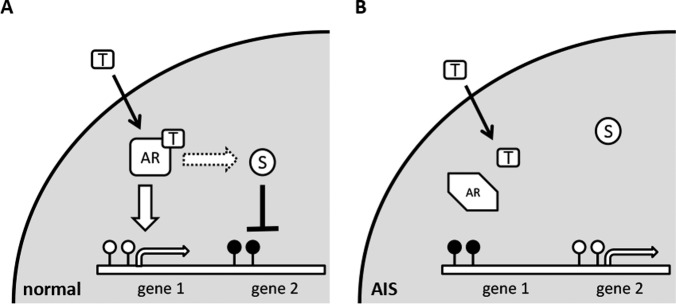
Model of establishment of DNA methylation patterns by AR activity. (A) Unmutated inactive androgen receptor (AR) binds testosterone (T) activating the receptor. Activated AR binds directly (large arrow) to AR response elements on the DNA inducing gene expression which subsequently prevents DNA methylation (“gene1”). Additionally either the activated AR itself or AR induced genes act on suppressor complexes (S; dotted arrow) which repress particular sets of genes (“gene2”) leading finally to DNA methylation of silenced genes. (B) In AIS missing AR activity prevents activation of AR target genes which might subsequently result to (stochastic *de novo*) DNA methylation of affected genes. In contrast, genes usually silenced by AR (directly or by additional AR-dependent pathways) become expressed preventing DNA methylation. white lollipops: unmethylated DNA, filled lollipops: methylated DNA.

Indeed, the *APOD* promoter contains a steroid hormone response element [45]–[47] and we have previously shown that APOD-mRNA is strongly androgen-inducible in normal male scrotum fibroblasts with intact AR but not in 46,XY CAIS - and PAIS labioscrotal fibroblasts [12]. Therefore, one might speculate that the diminished AR activation of *APOD* finally leads to its methylation in AIS. Figure 3 suggests a putative model how a defective AR pathway might finally result into an aberrantly shaped DNA methylome. Besides *APOD*, several additional AR target genes have been found to be aberrantly methylated in AIS as compared to male control cells (Table S2).

Moreover, the group of genes differentially methylated in AIS was enriched for low CpG content promoter genes. Such genes have also been found enriched in other non-malignant diseases [34]. In contrast, an enrichment of PRC2 target genes in embryonic stem cells which has been reported for several tumor entities [35], [48] has not been found in this study (data not shown).

Additionally we detected a statistically significant increase of stochastic variability of DNA methylation in a defined group of genes in AIS. This variability was significantly pronounced in the AIS patients as compared to male controls. Interestingly, in a current report Stadler et al. described that in a mouse model the interaction of transcription factors with the DNA is not only sufficient but moreover necessary to establish a distinct DNA methylation pattern [42], thus finally shaping the methylome at regulatory regions. This is in line with our findings suggesting that aberrant AR activity affects the DNA methylome. In AIS altered AR binding might directly affect the DNA methylation status of AR response elements (ARE). To a current model, DNA binding of transcription factors is required to etablish a regular DNA methylation pattern [42]. Besides a direct interruption of AR-binding, altered AR activity might interfere via signaling cascades also with the activity of genes and the load of proteins to regulatory sequences not containing AREs. One might speculate that in these cases protein binding to these DNA sequences is less efficient and sometimes might finally result into a disturbed DNA methylation pattern. We hypothesize that this mechanism could randomly modify the transcriptional background in target tissues. Accordingly, it may have roles in the variability and often unpredictability of the individual phenotype occurring in many monogenic diseases, particularly in AIS [49]. In this concept, the AR defect itself would always play the overriding functional role as a gate keeper. In case of complete inactivation of the AR, e.g., due to disruptive mutations in the *AR* – gene like a direct stop codon, the phenotype is invariably CAIS no matter of heterogeneity of gene methylation. This is confirmed by the CAIS patients in our study (e.g., ARD682, ARD1144, Table 1) in whom we have detected random methylation patterns but who have always normal female external genitalia. However, in partial AIS with preserved partial function of AR signaling (e.g., ARD527 [49]), a random methylation pattern may contribute to random transcriptional modifications giving rise to phenotypic heterogeneity. In contrast, a full functioning AR pathway in normal males will block random methylation supporting full male external genital differentiation. It is of interest in this respect, that many of the genes in the variability list – despite the fact that this list is not significantly enriched for AR targets - have documented roles in androgen−/genital tissue-related biology. These genes include (I) genes linked to spermatogenesis with e.g. *CAMK4* encoding calcium/calmodulin-dependent protein kinase IV [50] or *CA2* encoding carbonic anhydrase II [51] (II) genes associated with hypospadias like *IRF6* encoding interferon regulatory factor 6 [52] or (III) genes involved in prostate biology like *MGMT* encoding O-6-methylguanine-DNA methyltransferase [53] or *TMEPAI* encoding transmembrane prostate androgen-induced RNA [54]. In addition, this list includes genes associated with androgen function, including genes linked to androgen receptor co-regulators like *AATF* (Apoptosis-antagonising transcription factor) [55] or *CDC2L2* (cell division control like 2, CDK11-p58) [56], to androgen targets like *ALDH1A3* (aldehyde dehydrogenase 1A3) [57], *CA2*, *FMO2* (Flavin-containing monooxygenase) [58] or *TEMEPAI*, and eventually androgen programming (*HOXA5*) [59].

In summary, our data on a rare but exemplary human monogenic disorder of sex development suggest that abnormal AR-signaling in 46,XY individuals with AIS leads to significant changes in DNA methylation including a subset of genes with significantly increased heterogeneity of DNA methylation. In contrast, normal AR-signaling imprints a homogenous DNA-methylation, at least in genital fibroblasts. The observed methylation patterns are likely to be implemented prenatally when phenotypic genital sex is established. Our observations give rise to the concept that AR-mediated programming of methylation marks may play a relevant role in the establishment and maintenance of sexual dimorphisms in the human. In conclusion, androgen programming of the epigenome may be of general importance for the functional and phenotypic differences between males and females in health and disease.

## Supporting Information

Figure S1 **Unsupervised hierarchical cluster analysis and PCA of AIS samples and controls.** (A) Unsupervised hierarchical cluster analysis and (B) PCA of AIS samples (green bars/spheres) and controls (red bars/spheres). Only CpG loci annotated to AR target genes were included. A variance filter (σ/σ_max_>0.1) was applied; normalization: mean = 0, variance = 1. blue: low DNA methylation, yellow: high DNA methylation.
(TIF)

Figure S2 **Overview of the transcription start site of** ***APOD*** **as presented in the UCSC genome browser.** The position of the CpG locus hypermethylated in AIS is indicated. Furthermore, DNaseI hypersensitive sites and transcription factor binding sites identified by the ENCODE project are shown.
(TIF)

Figure S3 **Venn diagram of CpG loci characterized by hypermethylation, hypomethylation or high variability.** Venn diagram of CpG loci hypermethylated (“hypo”, n = 37) or hypomethylated (“hyper”, n = 130) in AIS as identified by differential methylation analysis and CpG loci characterized by a high variability in DNA methylation (“variable”, n = 79) as compared to the controls.
(TIF)

Table S1 Cell strains included in this study. CAIS: complete androgen insensitivity syndrome, female external genitalia.
(DOC)

Table S2 Loci aberrantly methylated in AIS compared to controls. List of genes and CpG loci aberrantly methylated in AIS as compared to normal controls. AR target genes identified using GATHER are indicated in blue.
(XLS)

Table S3 Loci characterized by high variation in DNA methylation patterns in AIS compared to controls. List of genes and CpG loci characterized by a high variation in the DNA methylation pattern in AIS as compared to normal controls (n = 75).
(XLS)

Methods S1 **Analysis of variability in the DNA methylation pattern.**
(PDF)
