# Supplementary figures and images for: Androgen Receptor Function Links Human Sexual Dimorphism to DNA Methylation
Source: PLoS One. 2013 Sep 4;8(9):e73288. doi: 10.1371/journal.pone.0073288 (PMC3762730; doi:10.1371/journal.pone.0073288)

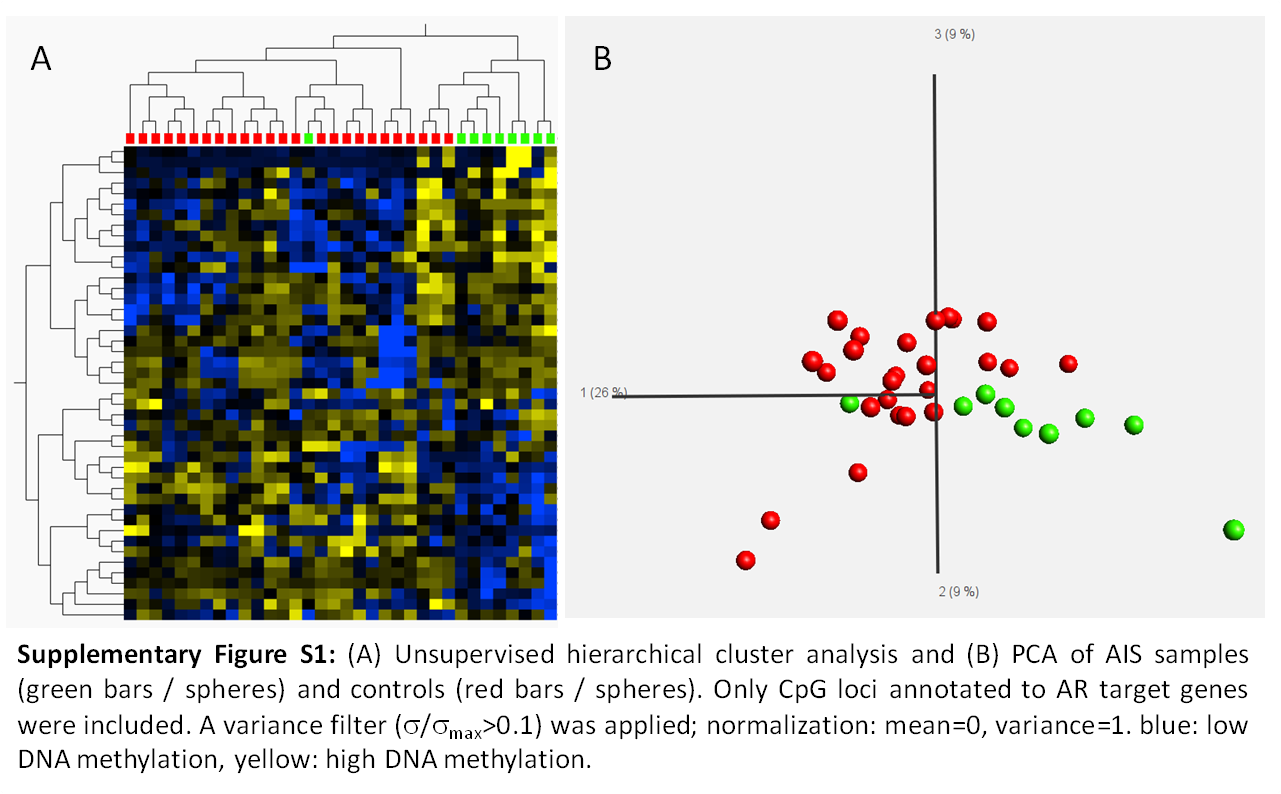

Supplement: Figure S1 — Unsupervised hierarchical cluster analysis and PCA of AIS samples and controls. (A) Unsupervised hierarchical cluster analysis and (B) PCA of AIS samples (green bars/spheres) and controls (red bars/spheres). Only CpG loci annotated to AR target genes were included. A variance filter (σ/σmax>0.1) was applied; normalization: mean = 0, variance = 1. blue: low DNA methylation, yellow: high DNA methylation. (TIF) [file pone.0073288.s001.tif]

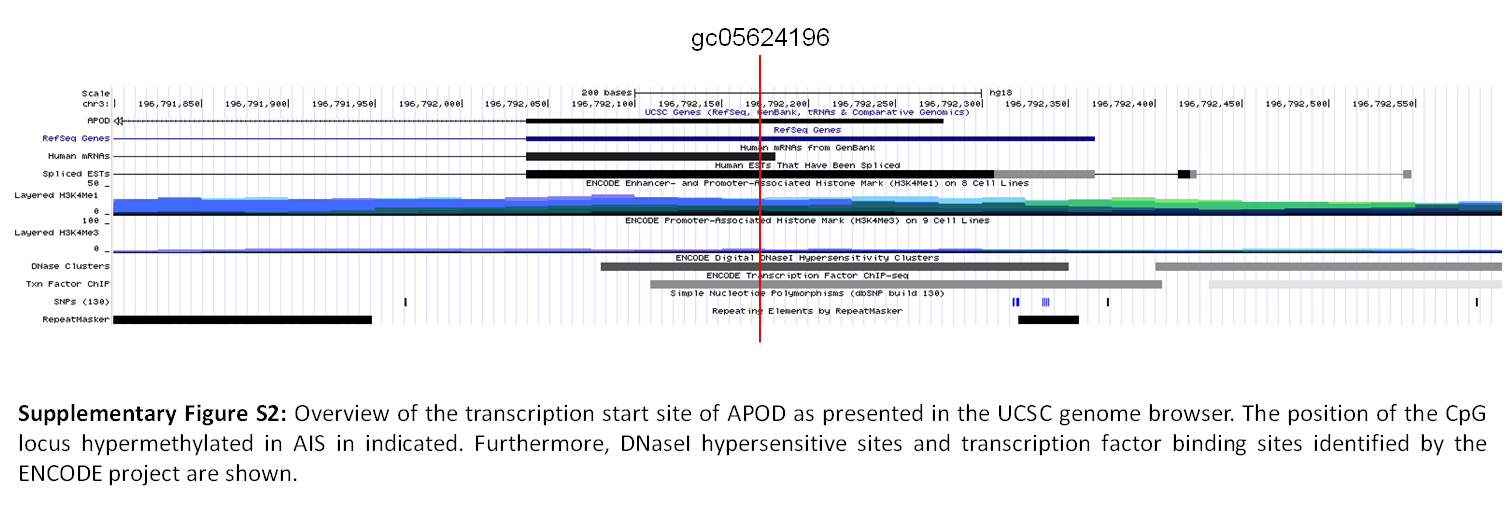

Supplement: Figure S2 — Overview of the transcription start site of APOD as presented in the UCSC genome browser. The position of the CpG locus hypermethylated in AIS is indicated. Furthermore, DNaseI hypersensitive sites and transcription factor binding sites identified by the ENCODE project are shown. (TIF) [file pone.0073288.s002.tif]

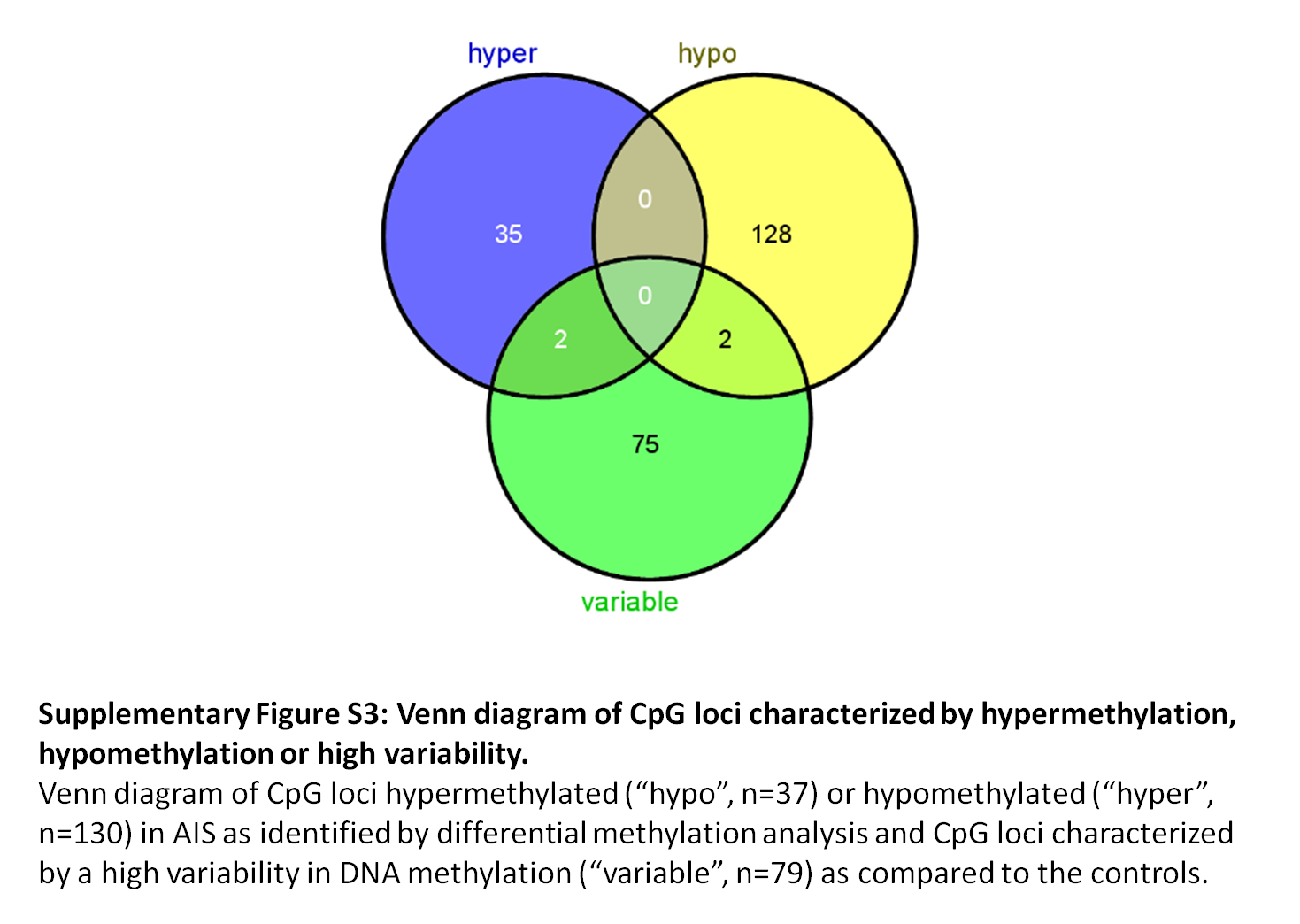

Supplement: Figure S3 — Venn diagram of CpG loci characterized by hypermethylation, hypomethylation or high variability. Venn diagram of CpG loci hypermethylated (“hypo”, n = 37) or hypomethylated (“hyper”, n = 130) in AIS as identified by differential methylation analysis and CpG loci characterized by a high variability in DNA methylation (“variable”, n = 79) as compared to the controls. (TIF) [file pone.0073288.s003.tif]
