# Supplementary material for: Androgen Receptor Function Links Human Sexual Dimorphism to DNA Methylation
Source: PLoS One. 2013 Sep 4;8(9):e73288. doi: 10.1371/journal.pone.0073288 (PMC3762730; doi:10.1371/journal.pone.0073288)
Supplement: Table S1 — Cell strains included in this study. CAIS: complete androgen insensitivity syndrome, female external genitalia. (DOC) [file pone.0073288.s004.doc]

**Table S1: Cell strains included in this study.** CAIS: complete androgen insensitivity syndrome, female external genitalia.

| **Cell strain** | **Phenotype** | **Age at biopsy (years;months)** | **AR mutation** |
| --- | --- | --- | --- |
| S8 | MAIS | 31;0 | Missense*^,#^ |
| C1282 | PAIS | 1;2 | Missense° |
| C1283 | PAIS | 1;2 | Missense° |
| 01RD0259 | PAIS | 17;2 | Missense |
| ARD 446 | AIS2 | 7;8 | Silent*^,#^ |
| ARD 527 | AIS2 | 1;6 | Missense^#^ |
| ARD306 | AIS2 | 7;8 | Missense^#^ |
| ARD 774 | AIS2 | 0;11 | Missense^#^ |
| 95E1022 | AIS2 | 6;4 | Missense |
| ARD 84 | AIS3 | 0;8 | None detected*^,#^ |
| ARD 1 | AIS3 | 0;9 | Splice site*^,#^ |
| 07E1268 | AIS3 | 1;6 | Missense |
| ARD 377 | AIS4 | 1;2 | Missense*^,#^ |
| ARD 659 | AIS4 | 3;10 | Missense*^,#^ |
| ARD 402 | CAIS | 1;0 | Frameshift*^,#^ |
| ARD 411 | CAIS | 0;4 | Missense*^,#^ |
| ARD 682 | CAIS | 14;10 | Stop*^,#^ |
| ARD 1097 | CAIS | 1;3 | Missense*^,#^ |
| ARD 1144 | CAIS | 4;3 | Stop*^,#^ |
| C872 | CAIS | 0;2 | Missense^ |
| C112 | CAIS | 1;10 | Frameshift |
| 84RD0200 | CAIS | 39;2 | Frameshift^$^ |
| 95RD0370 | CAIS | 4;10 | Missense^¶^ |
| 94RD0031 | CAIS | 2;5 | Splice site^§^ |
| 86E993 | CAIS | 0;1 | Stop |
| 94E366 | CAIS | 1;2 | Missense |
| S3 | male | 12;7 | None detected *^,#^ |
| S4 | male | 1;3 | None detected *^,#^ |
| S5 | male | 1;7 | None detected *^,#^ |
| S9 | male | 34;0 | None detected *^,#^ |
| S11 | male | 32;2 | None detected ^#^ |
| S12 | male | 9;2 | None detected *^,#^ |
| S13 | male | 58;1 | None detected * |
| S15 | male | 5;2 | None detected * |

AIS4: slight virilization, predominantly female external genitalia. AIS3: ambiguous external genitalia. AIS2: predominantly male external genitalia. MAIS: male external genitalia. PAIS: partial androgen insensitivity syndrome.* cases have been published in (a). ^#^cases have been published in (b). ^$^case has been published in (c). ^¶^ case has been published in (d). ^§^case has been published in (e). ^case has been published in (f).°cases have been published in (g).

1. Appari M, Werner R, Wünsch L, Cario G, Demeter J, Hiort O, Riepe F, Brooks JD, Holterhus PM (2009) Apolipoprotein D (APOD) is a putative biomarker of androgen receptor function in androgen insensitivity syndrome. *J Mol Med* 87(6):623-32
2. Holterhus PM, Deppe U, Werner R, Richter-Unruh A, Bebermeier JH, Wünsch L, Krege S, Schweikert HU, Demeter J, Riepe F, Hiort O, Brooks JD (2007) Intrinsic androgen-dependent gene expression patterns revealed by comparison of genital fibroblasts from normal males and individuals with complete and partial androgen insensitivity syndrome. *BMC Genomics* 18 (8):376.
3. Brüggenwirth HT, Boehmer AL, Verleun-Mooijman MC, Hoogenboezem T, Kleijer WJ, Otten BJ, Trapman J, Brinkmann AO (1996) Molecular basis of androgen insensitivity. *J Steroid Biochem Mol Biol* 58(5-6):569-75.
4. Boehmer AL, Brüggenwirth H, van Assendelft C, Otten BJ, Verleun-Mooijman MC, Niermeijer MF, Brunner HG, Rouwé CW, Waelkens JJ, Oostdijk W, Kleijer WJ, van der Kwast TH, de Vroede MA, Drop SL (2001) Genotype versus phenotype in families with androgen insensitivity syndrome. *J Clin Endocrinol Metab* 86(9):4151-4160.
5. Brüggenwirth HT, Boehmer AL, Ramnarain S, Verleun-Mooijman MC, Satijn DP, Trapman J, Grootegoed JA, Brinkmann AO (1997) Molecular analysis of the androgen-receptor gene in a family with receptor-positive partial androgen insensitivity: an unusual type of intronic mutation. *Am J Hum Genet* 61(5):1067-1077.
6. Jääskeläinen J, Deeb A, Schwabe JW, Mongan NP, Martin H, Hughes IA (2006) Human androgen receptor gene ligand-binding-domain mutations leading to disrupted interaction between the N- and C-terminal domains. *J Mol Endocrinol* 36(2):361-368.
7. Deeb A, Mason C, Lee YS, Hughes IA (2005) Correlation between genotype, phenotype and sex of rearing in 111 patients with partial androgen insensitivity syndrome. *Clin Endocrinol (Oxf)* 63(1):56-62.
