# Supplementary material for: Androgen Receptor Function Links Human Sexual Dimorphism to DNA Methylation
Source: PLoS One. 2013 Sep 4;8(9):e73288. doi: 10.1371/journal.pone.0073288 (PMC3762730; doi:10.1371/journal.pone.0073288)
Supplement: Methods S1 — Analysis of variability in the DNA methylation pattern. (PDF) [file pone.0073288.s007.pdf]

Supplementary material

Androgen receptor function links human sexual dimorphism to DNA methylation

Analysis of variability in the DNA methylation pattern

CpGs characterized by beta-values ranging in control samples up to 0.34 and differences of the interquartile differences above 0.16 between controls and AIS samples were considered as being variably methylated in AIS. These parameters have been deducted as follows:

CpG loci with beta-values ranged in control samples up to 0.34 and a difference of the interquartiles between controls and AIS samples above 0.16 were considered as being variably methylated in AIS and are included in this file.

These parameters were empirically determined from the following calculation based on the DNA methylation values:

$$n(k) = \sum_{j=0}^{m} (nc(j) - na(j))$$

k: looping variable ranging from 0.1 to 0.9

j: looping variable ranging from 0 to 0.3

m: m=30 (or m=j with na(j)=0, whatever is fulfilled first)

ΔIQ=|(Q.75(avg.beta[AIS])-Q.25(avg.beta[AIS]))-(Q.75(avg.beta[control])-Q.25(avg.beta[control]))|

nc= number of CpG loci which fulfill the following two conditions in the <u>control samples</u>:

1. max(avg.beta[controls])-min(avg.beta[controls])<k
2. ΔIQ >j

na= number of CpG loci which fulfill the following two conditions in the AIS samples:

1. max(avg.beta[AIS])-min(avg.beta[AIS])<k
2. ∆IQ >j

After plotting k against n(k) the apex has been determined at k=0.34 (plot A).

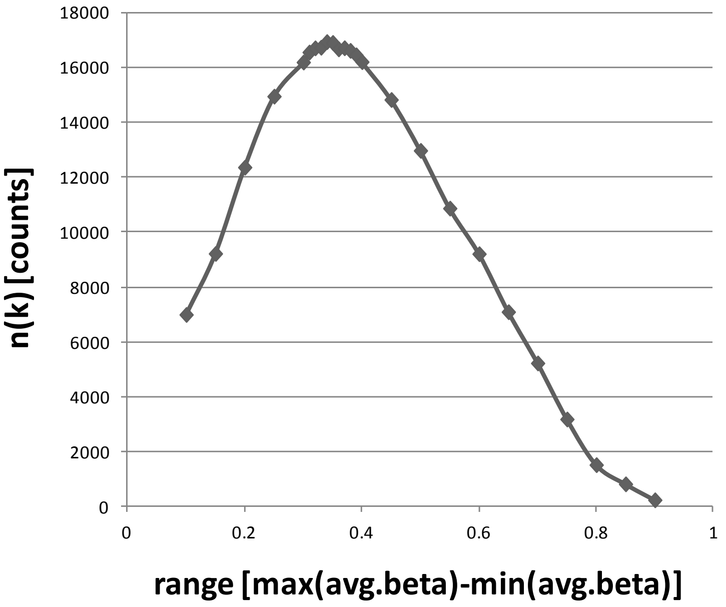

plot A: Determination of the maximal range (max[avg.beta]-min[avg.beta]) in controls to identify CpG loci characterized by high variability in their DNA-methylation values in AIS as compared to male controls.

To determine the ∆IQ for further analyses, the following calculation has been performed:

$$f(\Delta IQj) = \frac{nc(\Delta IQj) - na(\Delta IQj)}{nc(\Delta IQj)}$$

∆IQj: minimal interquartil difference (∆IQ) ranging from 0 to 0.26 (looping variable).

nc(∆IQj): number of CpG-loci with (Q.75(avg.beta[control])-Q.25(avg.beta[control])> ∆IQj and a maximum range (max[avg.beta]-min[avg.beta]) <0.34 (see above)

na(∆IQj): number of CpG-loci with (Q.75(avg.beta[AIS])-Q.25(avg.beta[AIS])> ∆IQj

After plotting ΔIQj against f(ΔIQj), the apex has been determined at ΔIQj=0.16 (plot B).

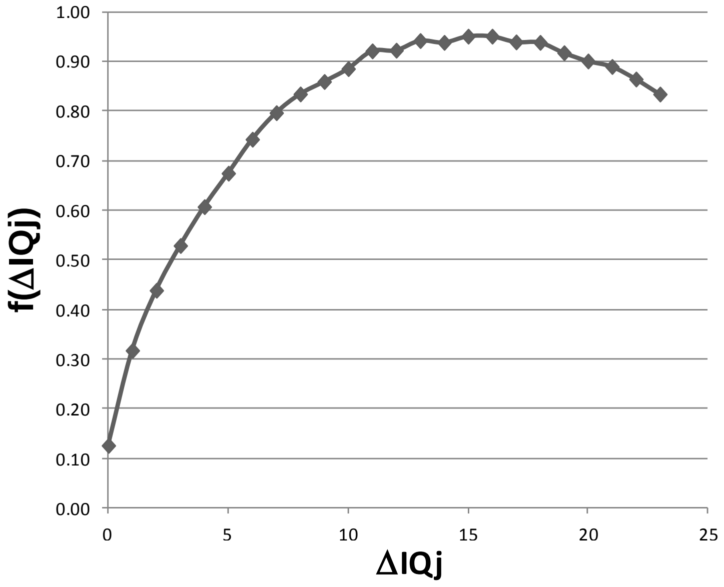

plot B: Determination of ΔIQ to identify CpG loci characterized by high variability in their DNA-methylation values in AIS as compared to male controls.

Microsoft Excel 2007 has been used for data visualization. Of 79 CpGs (75 genes) identified by this approach, 78 showed significant differences in variability ($p<0.05$, F-test). cg00532335, cg11471401, cg17706173, cg19485804 were also identified by the differential methylation analysis. Only 3 CpGs were identified (cg20537325, cg05520656, cg06908474) being more homogenously methylated in AIS than in controls.
